# Heartbeat detector from ECG and PPG signals based on wavelet transform and upper envelopes

**DOI:** 10.1007/s13246-023-01235-6

**Published:** 2023-03-06

**Authors:** Manuel Merino-Monge, Juan Antonio Castro-García, Clara Lebrato-Vázquez, Isabel María Gómez-González, Alberto Jesús Molina-Cantero

**Affiliations:** Departamento de Tecnología Electrónica, E.T.S.I. Ingeniería Informática, Universidad de Sevilla, Avd. Reina Mercedes s/n, 41012 Sevilla Sevilla, Spain

**Keywords:** ECG, PPG, Wavelet transform, Envelopes, Heartbeat detection

## Abstract

The analysis of cardiac activity is one of the most common elements for evaluating the state of a subject, either to control possible health risks, sports performance, stress levels, etc. This activity can be recorded using different techniques, with electrocardiogram and photoplethysmogram being the most common. Both techniques make significantly different waveforms, however the first derivative of the photoplethysmographic data produces a signal structurally similar to the electrocardiogram, so any technique focusing on detecting QRS complexes, and thus heartbeats in electrocardiogram, is potentially applicable to photoplethysmogram. In this paper, we develop a technique based on the wavelet transform and envelopes to detect heartbeats in both electrocardiogram and photoplethysmogram. The wavelet transform is used to enhance QRS complexes with respect to other signal elements, while the envelopes are used as an adaptive threshold to determine their temporal location. We compared our approach with three other techniques using electrocardiogram signals from the Physionet database and photoplethysmographic signals from the DEAP database. Our proposal showed better performances when compared to others. When the electrocardiographic signal was considered, the method had an accuracy greater than 99.94%, a true positive rate of 99.96%, and positive prediction value of 99.76%. When photoplethysmographic signals were investigated, an accuracy greater than 99.27%, a true positive rate of 99.98% and positive prediction value of 99.50% were obtained. These results indicate that our proposal can be adapted better to the recording technology.

## Introduction

The analysis of cardiac activity is one of the most common elements for evaluating the state of a subject [1, 2], whether to control possible health risks [3], monitor sports performance [4], determine the level of stress [5], etc. There are different noninvasive methods for recording cardiac activity, of which two of the most common are the electrocardiogram (ECG) and the photoplethysmogram (PPG). The information about a subject’s state of health provided by the ECG and PPG is not comparable. While the ECG details the electrical activity of the heart, the PPG records variations in blood volume as a result of cardiac activity. Thus, the ECG provides greater diagnostic value than the PPG. In the ECG, the morphology of the QRS complex makes it possible to identify certain cardiac pathologies [6, 7], but above all, as it is an important element in the ECG signal, its location helps to determine the position of other waves and segments of the ECG, such as the ST, whose level above baseline is associated with cardiac ischemia and myocardial infarction [8, 9]. Also, the analysis of the variability of the heart rate (HR), better know as heart rate variability (HRV), has been successfully employed in a multitude of situations, such as revealing diabetic neuropathies [10], analyzing the growth and condition of a fetus [11], or evaluating the regulation of cardiac activity by the autonomic nervous system, which facilitates the detection of possible pathologies in the latter [12]. In [13], the PPG was compared with the ECG to analyze heart rate variability. The results showed that the data from both techniques were very similar, so that PPG may be a valid alternative. However, when determining which technology to use to analyze cardiac activity, one has to consider the purpose of the recording, whether the analysis can be performed with ECG and/or PPG, as well as possible limitations, such as sensitivity to movement of the PPG [14, 15], the use of electrodes for the ECG, and the comfort of the individual during data collection. On this last point, the PPG may be more comfortable with respect to the ECG, as it is found in a large number of commercial devices, such as smartwatches, with which subjects are more familiar [16].

The process for heartbeat detection in ECG data usually has a first preprocessing phase to eliminate artifacts, followed by a candidate selection process that is used in the last phase to determine the temporal location of the heartbeats. To reduce the artifacts, that make it difficult to process the ECG data, a bandpass filter or an adaptive multiresolution technique -like wavelet transform- is usually applied, selecting frequencies in the range between [5, 20] *Hz*, which are associated with the QRS complexes [17–36]. The second stage generates a new signal in which the QRS complexes are enhanced. These heartbeat improvements can be accomplished by applying two different procedures that we named as type-A or type-B processes. The type-A processes consist of a signal rectification (the absolute value or the square of the data) followed by a sliding window integrator [17, 18, 20, 24, 26, 32, 36–38], and sometimes the Shanon energy is set in an intermediate stage [18, 24, 26, 31, 38], where QRS is accented with respect to the remaining signal elements by concentrating the energy around them, which is calculated as $-( d[n]^{2} log( d[n]^2 ) )$, where *d*[*n*] is the first derivative of the rescaled data between [0, 1]. As a result of the type-A process, the heartbeat appears as a kind of concave bell with a width approximately equal to the QRS-complex. The type-B processes are adaptive multiresolution techniques that decompose a signal into a set of signals with different frequency ranges associated with each one, so that temporal and frequency information is obtained simultaneously. Candidate selection is realized by choosing the levels to contain the frequency information associated with the QRS [20, 27, 39–41]. This way, type-A processes are simple and highly accurate, but sensitive to outliers, whereas type-B processes are more robust to outliers, but more complex. The final phase identifies the time points. This is typically accomplished by using fixed or adaptive thresholds. Hence, a heartbeat is associated with the time point at which these thresholds were exceeded [19, 20, 22, 25, 30, 33, 38, 42–44]. The accuracy of these techniques in determining the temporal location of the heartbeats is between 96.69% and 99.99%. Also, other authors use machine learning techniques on the preprocessed data to determine the location of the beats, eliminating the candidate selection phase [28, 45–47]. These studies have achieved an accuracy between 96.2% and 99.96% in locating heartbeats. Tables 1 and 2 summarize the main features of these studies. The databases Scopus^1^ and IEEE Xplore^2^ were used for this purpose.

**Table 1:** Main features of the selected manuscripts obtained through the review (part I)

References	Database	Main techniques	Accuracy (%)	Comments
[31]	MBA	WT; HT	99.83	
[39]	QT	Entropy; WT	99.85	11 records.
[17]	MBA	Pan-Tompkins process but different AT process.	96.26	48 records of 10 s. Positions of Q and S waves are calculated.
[40]	MBA	Non-Negative Matrix Factorization; AT	99.69	First channel of each ECG recording and excluded episodes of ventricular flutter from record 207.
[32]	MBA; NSR	WT; AT	99.99^B^	
[18]	MBA	BPF; SE; Digital first-order differentiator; HT	99.85	
[19]	12 database	BPF; Smoothing; EV	99.92	
[41]	MBA	Variable frequency complex decomposition method	99.89	
[33]	MBA; QT	EMD	99.89$^B$	Integrated P-wave detection.
[20]	MBA	BPF; HT; AT	99.50	
[45]	MBA; QT; CPSC20195	NN	99.96$^B$	
[21]	MBA	BPF; Variance; AT	99.69	
[22]	MBA	BPF; Exponencial Transform; AT	99.71	
[34]	QT; NST; INCART; CPSC2019^a^	WT; NN	97.48$^B$	ECG split into 10 s.
[43]	MBA	EV; AT	99.64	Integrated detection of P and T waves.
[46]	MBA; ESTT; INCART; MSA	NN	98.09	
[23]	MBA	BPF; MF	99.96	Integrated detection of P and T waves.
[24]	MBA	BPF; SE; HT	99.69	
[25]	MBA; QT	BPF; normalized cubic power; AT	99.84$^B$	Integrated detection of P and T waves.
[47]	QT; Lobachevsky	NN	96.90$^B$	Integrated detection of P and T waves.
[37]	MBA	Adaptative filter; AT	99.56	

By default, the databases are gathered from Physionet (https://physionet.org/)

*Acronyms: MBA* MIT-BIH arrhythmia, *NST* MIT-BIH noise stress test, *NSR* MIT-BIH normal sinus rhythm, *MSA* MIT-BIH supraventricular arrhythmia, *INCART* St Petersburg INCART 12-lead arrhythmia, *ESTT* European ST-T, *BPF* bandpass filter, *MF* median filter, *AT* adaptive thresholds, *HT* hilbert transform, *WT* wavelet transform, *EMD* empirical mode decomposition, *SE* shannon energy, *EV*  envelops, *NN* neural network, *PCA* principal component analysis

^a^Available in http://2019.cpscsub.com/

^B^The best case

**Table 2:** Main features of the selected manuscripts obtained through the review (part II)

Reference	Database	Main techniques	Accuracy (%)	Comments
[38]	Own data; QT; NST	EV; SE;	99.92$^B$	
[26]	MBA	BPF; SE; HT	99.86	
[27]	MBA	BPF; S-transform using zero-order prolate spheroidal wave functions	99.92	
[35]	MBA; INCART; Long Term ST; PTB Diagnostic	First derivative	99.82	Integrated detection and (on/off)set estimation of all ECG waves.
[28]	MBA; QT	Hierarchical clustering	99.83	Integrated detection and (on/off)set estimation of all ECG waves.
[36]	MBA	WT; MF; AT	99.62	
[44]	MBA; Fantasia	MF; Segmentation; AT; Statistical false peak elimination	99.72$^B$	
[29]	QT	EMD; BPF	99.90	
[30]	MBA; NST; ESTT; QT	Nonlinear filter; AT	99.98^B^	

By default, the databases are gathered from Physionet (https://physionet.org/)

*Acronyms: MBA* MIT-BIH arrhythmia, *NST* MIT-BIH noise stress test, *NSR* MIT-BIH normal sinus rhythm, *INCART* St Petersburg INCART 12-lead arrhythmia, *ESTT* European ST-T, *BPF* bandpass filter, *MF* median filter, *AT* adaptive thresholds, *HT* hilbert transform, *WT* wavelet transform, *EMD* empirical mode decomposition, *SE* shannon energy

^B^The best case

One of the most widely used algorithms to detect QRS complexes is the pan-tompkins (PT) [48]. It applies adaptive thresholds to the output resulting from integrating a 150 ms sliding window over the square of the derivative of a bandpass filter’s output. The accuracy of this technique is better than 99.3% [48]. Other techniques employ wavelet transform, envelopes and/or some classification technique, such as k-means, to detect QRS complexes [49, 50]. For example, in [49], envelope-based filtering is used to isolate QRS complexes, followed by a k-means classifier to differentiate between QRS and other spikes (hereafter, we refer to this technique as EK). The lower/upper envelopes are the result of a process that (1) locates the minima/maxima and (2) finds the interpolation curve passing through them [51]. This way, EK obtains the average of two lower envelopes twice to remove non-QRS-complex waves. In [50] is presented a modified version of PT, where the derivative of the bandpass filter output is replaced by the stationary wavelet transform, all other components being equal, so that it employs the same adaptive thresholding system (hereafter, we will refer to this technique with the acronym SWT). In this one, applying the wavelet transform (WT), data is decomposed into 2 levels using a Daubechies mother wavelet with vanishing moments of 3. Level 2 coefficients correspond to the frequency band of QRS complexes. In this way, both algorithms achieved an accuracy of better than 99.7%.

**Fig. 1:**
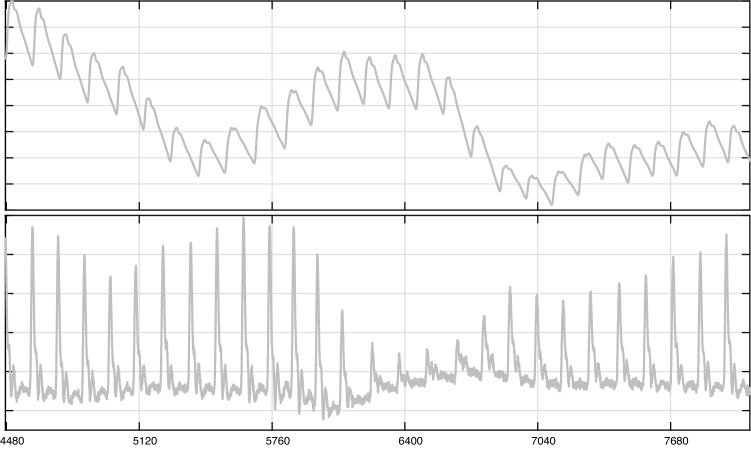
Recording using PPG measures in participant’s thumb (sampling rate of 128 Hz from DEAP dataset—Sect. 2.2). The signal of PPG is shown above, and its first derivative (dPPG) is shown below. The x-axes are set in samples

PPG signals are usually processed in 2 stages as shown in [52–55], where the accuracy was reported to be between 85% and 97.67%. In the first one, the signal noise is reduced by a 0.6–5 Hz bandpass or a median filter, while in the second one, the peaks from the smoothed PPG signal or its first derivative are located. Applying the first derivate to PPG (dPPG) [56] it is possible to obtain a signal with concave-bell-shaped waveform segments, similar to the QRS complex (Fig. 1), so techniques for detecting QRS in ECG signals are potentially applicable to PPG data. The techniques described above are mainly focused on ECG signals, so their performance with PPG signals has not been evaluated (no study from January 2017 to July 2022 has performed this analysis). In turn, the adaptive thresholds may present difficulties in adapting to an abrupt drop in amplitude, as occurs in the QRS or in the dPPG signal (Fig. 1), which may cause some QRS to go undetected. The abovementioned underlines the need to establish a process for determining the locations of heartbeats automatically and reliably in both recording techniques.

## Methodology

In this paper, we present a technique to detect heartbeats in ECG and PPG signals, which is explained in detail in the next section, along with the databases and analysis performed.

### Heartbeat detection using wavelet transform and envelopes

**Fig. 2:**
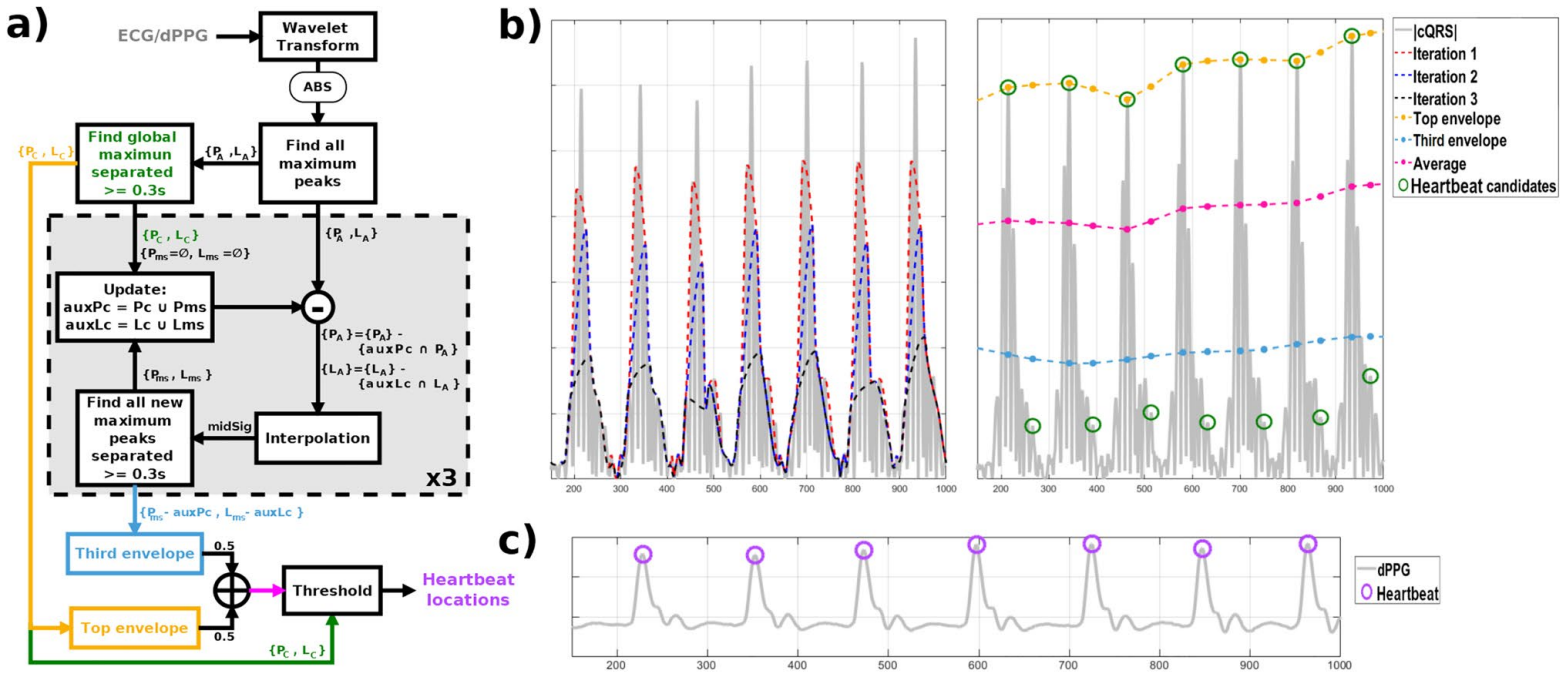
Summary of our proposal. Example of PPG signal processing with our proposal for a subject from DEAP dataset with sampling rate of 128 Hz and amplitude range of [− 262.144, + 262,144] mV. The x-axes are set in samples. **a** Schematic of the proposed technique. **b** The different phases of the algorithm: absolute value of the output of the MOWT ( $\mid cQRS \mid$), the selection of peaks and the resulting envelopes. On the left, the iterated process to obtain the envelopes, and on the right the envelopes and the QRS candidates. **c** The derivative of the PPG with the location of the QRS that provides the output of our proposal

To locate heartbeats in ECG and dPPG signals, we propose a new technique based on maximal overlap discrete wavelet transform and upper envelops (WE) (Fig. 2). The maximal overlap discrete wavelet transform (MOWT) aims to select the peaks with the highest amplitude of the signal, while the QRS complex, and its position, is associated with the peak of the signal that exceeds the average of the envelopes. The envelopes result from locating all local maxima or minima and interpolating a signal passing through all of them [51]. If these peaks are maxima, the result is the upper envelope, while if they are minima, the lower envelope is obtained. Thus, the process is divided into 3 parts: (1) heartbeat enhancement using the MOWT, (2) calculation of the local maxima, (3) obtaining the envelopes and localization of the QRS complexes. The “Appedix A” shows the pseudocode of our proposal.

#### Heartbeat enhancement

MOWT divides a signal into different components with temporal and frequency information, which makes it easy to detect from QRS complexes. Three fundamental aspects should be considered to select QRS complex candidates:

1. The *wavelet mother* to be used. The more this resembles the QRS complex, the better the decomposition. For that reason, we selected the Symlets wavelet with 4 vanishing moments. This is a nearly symmetric variation of the Daubechies family of wavelet mother, and its resemblance to the QRS complex ensures a correct decomposition of the data.
2. The number of components, *N*, into which the ECG signal is to be decomposed. Since the sampling rate may vary from one capture to another, the number of components is set as the value resulting from taking the integer part of the logarithm in base 2 of the sampling rate, that is, $N=\lfloor log_2( F_s ) \rfloor$.
3. The frequency range associated with QRS complexes is in the interval [5, 20] Hz. Thus, those levels of the MOWT decomposition that contain this interval will be chosen. The remaining ones are discarded.

After the previous steps, we proceed to calculate the modulus of the inverse transform of the MOWT, obtaining a signal with the possible QRS candidates, which we will call from now on $\mid cQRS \mid$ (Fig. 2b).

#### Local maximums

The search for local maxima is performed on $\mid cQRS \mid$. This process is iterated, with the aim of obtaining a signal that dynamically adapts to the temporal evolution of the QRS complexes and allows them to be detected (Fig. 2b). The steps are as follows:

1. Let $P_A$ be the set of the magnitudes of all local maxima present in $\mid cQRS \mid$ and $L_A$ be the set containing their temporal locations in the signal.
2. Let $P_C$ and $L_C$ be the sets containing the local maxima of $P_A$ and their time positions that are more than 300 ms apart. This is done by applying an iterated procedure consisting of choosing the highest peak among all the candidates and eliminating all those that are less than 300 ms away from it. This is then repeated for the highest of the remaining peaks, and reiterated until there are no candidates left.
3. Let $aux_{Pc}$ and $aux_{Lc}$ be a copy of $P_C$ and $L_C$ respectively. With those copies, we proceed to execute the following procedure 3 times (Fig. 2b, left):
  - All elements in $aux_{Pc}$ and $aux_{Lc}$ are removed from $P_A$ and $L_A$.
  - A new signal, *midSig*, of equal length to $\mid cQRS \mid$, resulting from interpolating all remaining local maxima in $P_A$ is generated. This interpolation is based on a piecewise cubic Hermite interpolating polynomial.
  - Let $P_{ms}$ and $L_{ms}$ be all local maxima of *midSig* that are at least 300 ms apart (as explained above), discarding those peaks that are in $aux_{Lc}$.
  - The maximums resulting from the previous step are added, in temporal order, to the variables $aux_{Pc}$ and $aux_{Lc}$.

After finishing the previous procedure, the variables $P_C$, $L_C$, $P_{ms}$ and $L_{ms}$ will store the peaks that will be used to generate the envelopes.

#### Envelopes

From the above peaks, we compute two upper envelopes: one centered on the variables $P_C$ and $L_C$, and another based on $P_{ms}$ and $L_{ms}$ and passing through the positions stored in $L_C$. These are obtained by finding the local maxima of the variables $P_C$ and $P_{ms}$, and interpolating a signal passing through these points using a piecewise Hermite cubic interpolation polynomial (Fig. 2b, right). From the envelopes, the average of both is calculated, so that all $P_C$ peaks above this average will be classified as a heartbeat.

#### Online implementation

**Fig. 3:**
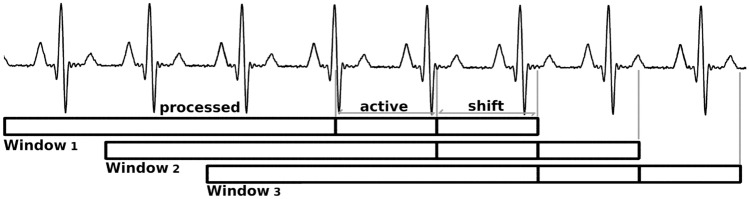
Online-version buffer. Buffer length of 7.5 s, and *active* and *shift* areas of 750 ms

The implementation of our proposal to detect QRS complexes in real time requires the establishment of a data window as well as a shifting between windows (Fig. 3). To ensure that the window contains enough data, such that the envelopes match the variations of the QRS complex amplitudes, a length of 7.5 s and a shift of 750 ms have been set, so that if an individual has 40 beat per minutes (BPM), the window will contain at least 5 QRS complexes. The data buffer is divided into 3 areas: *processed*, *active* and *shift*. The process described above is applied to the entire window, so that only the QRS detected in the *active* zone will be taken into account. The *processed* and *shift* zones ensure that the envelopes are properly adjusted to the amplitudes of the QRS complexes. The length of the *active* and *shift* zones are identical, being set at 750 ms, ensuring that QRS is detected in subjects with a minimum of 40 BPM (1.5 s between beats), which minimizes the possibility of loss of complex detection.

### Heartbeat database

**Table 3:** Heartbeat databases

Database	Description	Subjects	Signals	Hearbeats	Sampling rate (Hz)	Time (s)
European ST-T	70 men and 8 women. 24 h Holter recording from ambulatory ECG data. Analysis of ST and T-wave changes.	e0106	V3	16,443	250	20,000
		e0103, e0104, e0105	V4			
Fantasia	40 healthy subjects (20 men and 20 women). 120 min recordings of ECG and respiration in supine rest.	f1y10, f2y07, f2o06, f2o10	ECG1	16,604	250	16,000
MIT-BIH arrhythmia	48 half-hour excerpts of ambulatory ECG data. 47 individuals. arrhythmia analysis.	100	V5	8493	360	7220
		100,101, 103	MLII			
MIT-BIH normal sinus rhythm	5 men and 13 women. Normal sinus rhythm (no significant arrhythmias). ECG recordings obtained from 24 h sessions.	16,265, 16,272, 16,273, 16,420	ECG1	40,595	128	31250
MIT-BIH supraventricular arrhythmia	78 half-hour ECG data from long-term Holter records. supraventricular arrhythmias	800, 802, 803	ECG1	5622	128	5400
DEAP dataset	32 volunteers watched 40 music videos of 63 s each one. PPG data recorded from left thumb.	s01–s22	PPG	61,456	128	55,440

First column: database name; second column: basic database information; third column: id of individuals in data bases; fourth: total number of heartbeats in each database; fifth column: the sampling frequency of each database; sixth column: total time of analyzed data in each database

The data used in this study were obtained from PhysioNet databases (PDB) [57–61] and DEAP dataset (DEAP) [62] (Table 3 shows the detail of the databases used). PDB contains signals from ECG whose selection was made based on [49]. In that article, the signals were randomly selected to analyze the effectiveness of the algorithm against different morphologies of QRS complexes. Specifically, three of the databases contained cardiographic recordings from subjects with various heart diseases, while the other two selected were from healthy subjects. In total, we analyzed 22 h and 11 min of data with expert annotations, which we used to compare the QRS detected by our proposal and the other techniques using an automated process. PDB provides the data with the power grid interference already removed. In DEAP, the cardiac activity was recorded using PPG, from which, using the dPPG, data resembling the ECG signals were obtained. A low-pass filter with a cutoff frequency of 30 Hz was applied to this signal, whose output was used as input to the different detectors. From the DEAP database, subjects 23 to 32 were discarded for having incorrect records. A total of 15 h and 24 min were analyzed manually, since the database lacked annotations with the locations of the heartbeats. Finally, it should be noted that no additional processing was applied to reduce noise, except for those already mentioned.

### Evaluation and statistical analysis

Our proposal is compared with 3 techniques were described in Sect. “Introduction”: PT [48], EK [49], and SWT [50]. The first was selected because it is one of the most cited techniques; the second was used to select the ECG databases and uses envelope calculation as its main core; the third was selected because it uses the wavelet transform and is implemented in the *py-ecg-detectors*^3^ package for Python [63]. All the techniques were implemented, simulated and analyzed with Matlab version 9.12.0.1975300 (R2022a).

The analysis of our proposal assesses its effectiveness and efficiency, while comparing it with the three algorithms mentioned. To do so, we counted how many beats were correctly detected—true positive (TP), how many were incorrect—false positive (FP), and how many were not detected—false negative (FN). With this information, we calculated: (a) true positive rate (TPR), which measures the probability that a true QRS complex is correctly detected; (b) positive prediction value (PPV), which gives the probability that the positive results of the processing are true QRS complexes; (c) accuracy (ACC), which refers to the overall performance of the algorithm. The Eqs (1–3) show how these parameters are calculated. In addition, the Wilcoxon signed-rank test was applied to these three parameters to verify whether the differences of our proposal with respect to the others were due to random chance. For each technique, three vectors were obtained with the values of TPR, PPV and ACC. The *p*-value of the statistical test was obtained taking our proposal as a reference.

1 $$\begin{aligned} TPR= & {} 100 \cdot TP / (TP + FN) \end{aligned}$$

2 $$\begin{aligned} PPV= & {} 100 \cdot TP / (TP + FP) \end{aligned}$$

3 $$\begin{aligned} ACC= & {} 100 \cdot TP / (TP + FP + FN) \end{aligned}$$

## Results

The results obtained after applying the techniques to the databases are shown in the Table 4. For ECG data one can see that all the algorithms present very similar values, with PT, and our proposal, being the ones with the best results according to the three evaluated indicators (TPR, PPV and ACC are greater than $>99.93$%), although without significant statistical differences between all techniques, except with EK. The PT algorithm obtained a slightly higher TPR but lower PPV and ACC with respect to our proposal. On the other hand, the analysis of the data recorded by PPG shows that our technique was superior to the others, both in terms of accuracy, ability to detect QRS complexes and reliability (TPR, PPV and ACC are greater than $>99.27$%), with this difference being significant except for PPV with respect to the data from PT and SWT. Furthermore, statistical analysis of the combined PPG and ECG results also shows that our technique performs significantly better than the others.

**Table 4:** Results of QRS detectors

	PPG						ECG						PPG + ECG		
	TP	FP	FN	TPR	PPV	ACC	TP	FP	FN	TPR	PPV	ACC	TPR	PPV	ACC
**WE**	61387	289	69	**99**.**887**	99.531	**99**.**420**	87737	13	35	99.960	**99**.**985**	**99**.**945**	**99**.**930**	**99**.**798**	**99**.**729**
**RWE**	61313	304	143	99.767	99.507	99.276	87739	16	33	99.962	99.982	99.944	99.882	99.786	99.668
PT	61073	1013	383	99.377$$^{***}$$	98.368	97.765$$^{***}$$	87740	22	32	**99**.**964**	99.975	99.939	99.722$$^{***}$$	99.309	99.035$$^{***}$$
EK	59668	243	1788	97.091$$^{***}$$	**99**.**594**$$^{***}$$	96.708$$^{***}$$	87717	148	55	99.937	99.831$$^{*}$$	99.769$$^{**}$$	98.765$$^{***}$$	99.735$$^{***}$$	98.507$$^{***}$$
SWT	59463	1570	1993	96.757$$^{***}$$	97.428	94.347$$^{***}$$	87713	21	59	99.933	99.976	99.909	98.625$$^{***}$$	98.931	97.585$$^{***}$$

The first two groups show the results of each type of signal individually. In the last group, PPG + ECG, the previous results are put together to analyze the overall performance of the techniques. Algorithms employed: WE - our proposal; RWE - online implementation of WE; PT [48]; EK [49]; SWT [50].

Results of Wilcoxon signed-rank test with respect to WE: *p-value $< 0.05$; **p-value $< 0.01$; ***p-value $< 0.001$

## Discussion

The proposed technique demonstrated a high level of accuracy in the detection of heartbeats for ECG and PPG signals, which implies more independence with respect to the method used for data recording. Both the overall results yielded from the offline version, as well as from its real-time implementation, were high ($>99.66$%). These values were higher than those obtained by the other algorithms with which they were compared. These differences were mainly due to the PPG, whose signal had first to be derived, and then have a 30 Hz low-pass filter applied, to obtain waves similar to the QRS complexes of the ECG. In this way, we were able to apply the same algorithms as for the localization of the QRS in ECG. However, the amplitudes of these complexes in the PPG showed greater variability with respect to the ECG data. This variability makes the EK technique performs worse performance in comparison with our proposal, since it uses lower envelopes to isolate the QRS from the remaining components of the ECG signal and other noises. Thus, if a QRS has variations in its peak, so that it presents local minima, the lower envelope process will cause the QRS amplitude to decrease or be eliminated. On the other hand, the PT and SWT algorithms employ the same adaptive thresholding process. This results in certain QRS complexes with a much smaller amplitude not being detected. Finally, in the SWT technique, in addition to the aforementioned thresholding problem, a higher occurrence of FP is added due to the oscillations resulting from applying the stationary wavelet transform. Thus, our proposal, which uses envelopes as adaptive thresholds to detect QRS complexes, shows greater accuracy by adapting appropriately to the temporal evolution of QRS amplitudes.

The TPR of the online version of our proposal, applied to the ECG, was slightly higher with respect to the offline version, while its PPV was slightly lower. These small differences were due to the fact that in the offline version, the envelopes were globally adjusted to the totality of the QRS, while in the online version the adjustment was by smaller sections which included a few complexes. This caused some QRS misclassified in the offline version to be detected in the online version, but in turn this caused the online version to generate more FP. In both implementations, some of these errors occurred at the beginning and end of the data vector, since the fit at these ends was not correct. A possible solution could be to check whether the amplitude of the first and last QRS complexes differ by 50% in amplitude with respect to the posterior and anterior, respectively, and discard them if the difference is greater than 50%. However, we believe that this is not a significant problem because it is located at the beginning and end of the data.

On the other hand, the offline version requires 2 parameters which are: (a) the sampling rate and (b) the distance in seconds between local maxima. The online version needs 2 additional parameters: (a) the buffer length and (b) the shifting length. Focusing on the sampling rate, the MOWT is used to select the frequency range of [5, 20] Hz. This assumes that the minimum sampling rate must be 40 Hz, and the minimum data time to have a spectral resolution of 5 Hz at this sampling rate is 200 ms. For there to be at least one heartbeat in this time, the HR must be at least 300 BPM, which implies ventricular fibrillation. Therefore, in general, the minimum data length needs to be greater than 200 ms, which ensures sufficient spectral resolution to select the frequency interval associated with the QRS complexes. With respect to the second parameter, the temporal distance of 300 ms between consecutive QRS candidate peaks is a factor that limits the applicability of our technique, since if the HR is greater than 200 BPM, some QRS complexes may not be detected. However, HR above this value are infrequent, around 35 per 100,000 patients per year [64], so we consider this limitation to be uncritical. Regarding the parameters of the online version, the proposed buffer length can be reduced if memory requirements so require. Taking as a reference the 200 BPM, a value well above the normal resting range [65], the *shift*, *active* and *processed* zones must have a duration of at least 300 ms for the buffer to contain at least 2 QRS complexes, so the window would be 900 ms. Likewise, to detect a minimum HR of 40 BPM, the buffer should be at least 3 s and the shifting 750 ms. Another factor affected by the buffer length is system delay in providing the location of the QRS complexes. For the values set in the Sect. 2.1.4, the time it takes to give the first data is 7.5 s, and then 1.5 s, which can be reduced if necessary as indicated above. Additionally, the PPG signal has an intrinsic delay of up to 250 ms due to its dependence on blood pressure [66], resulting in a higher variability that must be taken into account when used for monitoring individual. These values are acceptable in a multitude of situations such as day-to-day activities, physical exercise, hospital monitoring, etc.

As mentioned above, the PPG can be recorded on various areas of the body [67], so in certain situations where subject comfort is sought, and where noise due to movement is minimal, the PPG may be preferred over the ECG. Also, people tend to be more familiar with the idea of wearing a watch-like device [16], which could be a key factor in selecting which technology to use. On the other hand, the reuse of modules and code across manufacturers reduces the expense of product development [68]. As our proposal is highly reliable in both ECG and PPG signals, it enables the reuse of code and/or modules at manufacturers, reducing development cost. At the same time, it facilitates decision-making at the level of scientific and/or medical research, since the analysis of the variability of the HR is carried out with guarantees with both recording technologies.

Finally, our proposal presents a first stage for highlighting QRS complexes followed by an adaptive threshold, like most of the techniques contained in Sect. 1. However, the first stage does not seek to generate a concave bell that covers the QRS after rectification of the MOWT, but rather to obtain peaks that allow the threshold to be adjusted to the variation in QRS amplitudes and thus improve the localization of the QRS. Thus, the results obtained by our proposal are superior to the majority of the articles included in the Sect. 1 when used in ECG signals (Tables 1 and 2). They are located in the first quartile, so that only 4 of them have a higher accuracy [23, 30, 32, 45]. However, none of these reflect their effectiveness on data derived from the PPG signal, so we cannot establish a comparison with our proposal.

## Conclusion

In this work we have described an algorithm applicable to ECG and PPG signals, with a high accuracy in detecting heartbeats due to the use of envelopes as an adaptive threshold, as these are better adjusted to the variability of the amplitude of QRS complexes. For offline version, the accuracy in detecting such QRS was higher than 99.94% for ECG and 99.42% for PPG, with a high probability of detecting true QRS complexes (TPR $>99.8$% for PPG and $>99.96$% for ECG) and a high reliability that the processing outputs were real QRS complexes (PPV $>99.50$% for PPG and $>99.98$% for ECG). On the other hand, the online version was slightly better at detecting QRS in ECG signals, but at the cost of a higher number of FP.

The analysis of cardiac activity is used in multiple applications: processes to detect pathologies, in the calculation of caloric expenditure, analysis of possible health risks during sports activities, in systems that seek to determine the emotional state of a subject, stress, cognitive load, etc. Therefore, the applicability of our proposal is evident, as it is flexible with respect to the data recording technique and highly accurate in the localization of QRS complexes.

## Appendix A

### Pseudocode of our offline proposal

To improve the understanding of the technique developed in this paper, the pseudocode that implements the offline version of our proposal is shown below.

## Footnotes

1 https://www.scopus.com.

2 http://ieeexplore.ieee.org.

3 https://github.com/berndporr/py-ecg-detectors
